# Dementia in a Black and minority ethnic population: characteristics of presentation to an inner London memory service

**DOI:** 10.1192/pb.bp.114.047753

**Published:** 2015-08

**Authors:** Rosalyn Tuerk, Justin Sauer

**Affiliations:** 1 South London and Maudsley NHS Foundation Trust, London UK; 2 Institute of Psychiatry, King's College London

## Abstract

**Aims and method** To examine data on referrals to an inner-city London memory service to explore any differences in referral rates, cognitive assessments and stages of dementia at presentation between ethnic groups.

**Results** African–Caribbean patients were well represented in the memory service. They were diagnosed with dementia on average 4.5 years younger than their White British counterparts and were more likely to be diagnosed with a vascular or mixed type dementia. However, scores on initial cognitive testing were significantly lower in the African–Caribbean group, possibly representing more advanced disease at presentation.

**Clinical implications** Initiatives to access Black and minority ethnic populations earlier in the course of their illness should be considered. Professionals need to consider the potential for cultural bias in memory testing and diagnosing dementia in these populations, and the importance of cultural competency in assessments.

The number and proportion of Black and minority ethnic (BME) people with dementia is set to increase sharply over the coming years.^1^ As the ‘most ethnically diverse area’ across England and Wales,^2^ memory assessment services in London must be able to meet this challenge and address the needs of people from BME backgrounds. Established in 2010, the Southwark and Lambeth Memory Service (SLMS) provides memory assessment services in two inner London boroughs, to people over the age of 18 with mild to moderate memory problems and without an existing diagnosis of dementia. Patients are offered a comprehensive assessment, with initial cognitive testing using the Standardised Mini-Mental State Examination (SMMSE) and Addenbrooke's Cognitive Examination III (ACE-III). Patients are also offered brain neuroimaging and, where indicated, additional neuropsychological testing. Diagnoses are considered within a multidisciplinary team and appropriate treatment and follow-up are agreed with patients and carers.

The National Dementia Strategy^3^ prioritises early diagnosis and intervention in people with dementia. Current research, however, suggests that people from BME backgrounds present to memory assessment services later in the course of their disease.^4^ This prevents them from benefiting from intervention and treatment as early as their White British counterparts.^5^ This study examines the stage at which the BME population presents to SLMS and their rates of dementia diagnosis, and considers subsequent areas for service development to better meet the needs of different ethnic groups.

## Method

The study received approval from the shared research and development office of the Institute of Psychiatry and Maudsley Hospital. The analysis used anonymised data from a referrals database, maintained for the purposes of service monitoring, and includes patients referred during the first 2 years of the SLMS's operation. Information on patients' ethnicity, age at the time of referral, scores on initial cognitive testing and the diagnosis given by the service was collated. Data are drawn from the SLMS's use of earlier versions of the cognitive tests cited above (the Mini-Mental State Examination (MMSE)^6^ and Addenbrooke's Cognitive Examination Revised (ACE-R)^7^). In cases where patients' diagnostic classification changed during the course of their involvement with the service (e.g. from mild cognitive impairment to dementia), the initial diagnosis is used. To avoid bias, people unable to fully complete the two cognitive tests were excluded from the analysis.

We used the harmonised ethnic group categorisations from the 2001 UK census^8^ to allow for comparisons between the SLMS sample and data on the ethnic composition of the local population. For the purpose of data analysis, patients were divided into two groups: White British (WB) and Black and minority ethnic (BME). Definitions of BME are wide-reaching and may include anyone with a ‘cultural heritage which is differentiated from that of the White majority of the UK’.^9^ Owing to the very small number of patients from some ethnic groups in the SLMS sample, only patients from Caribbean and African backgrounds were included in the BME group. Comment is made in the discussion section on the relevance of the findings to people from other BME backgrounds.

Differences in the stage of presentation to the memory service were analysed by comparing patients' ages at the time of referral and MMSE and ACE-R scores on initial cognitive assessment. The prevalence of dementia diagnoses within each of the two groups was compared using Pearson's chi-squared test. Scores on cognitive testing for patients receiving a diagnosis of dementia were also compared. Unless otherwise stated, all means were compared using Student's *t*-test, having first satisfied the criteria for Levene's test of equality of variances; *P*=0.05 were deemed to be significant.

## Results

Overall, 460 patients referred between January 2011 and December 2012 completed the assessment process and received a diagnosis from the service. Of these, 384 were from White British, Black Caribbean and Black African ethnic groups (WB: *n* = 239, 68.7%; BME: *n* = 109, 31.3%). Then, 290 patients completed cognitive testing in full; proportions of patients from each group able and/or willing to complete full cognitive testing were similar (WB: *n* = 199, 83.3%; BME: *n* = 91, 83.5%).

The majority of SLMS patients were over 60 years of age. Table 1 compares the most common ethnic groups within the SLMS sample and the local population of people over the statutory retirement age. These are notably different in distribution, with the SLMS sample comprising a smaller percentage of White British patients and larger proportions of the other featured ethnic groups.

**Table 1:** Comparison of most common ethnic groups in the SLMS sample and local population

Ethnicity	Patients in SLMS sample (*n*)	Percentage of total SLMS sample^a^	Local population over 60 years of age (*n*)^10^	Percentage of local population^a^
White: British	239	52.0%	36 100	63.4%

Black or Black British: Caribbean	86	18.7%	6300	11.1%

White: any other white background	47	10.2%	3500	6.2%

White: Irish	38	8.3%	3800	6.7%

Black or Black British: African	23	5.0%	2400	4.2%

Asian or Asian British: Indian	12	2.6%	1300	2.3%

All other BME groups	15	3.3%	3500	6.2%

SLMS, Southwark and Lambeth Memory Service.

a. Percentages may not add up to 100 owing to rounding.

At referral to the service, BME patients (mean age 77.19 years, s = 7.094) were significantly younger than WB patients (mean age 80.23 years, s = 8.777; *t*(212.729) = 3.132, *P* = 0.002). In this case, Levene's test could not conclude equality of variances (*P* = 0.026), therefore a *t*-test appropriate to samples with potential unequal variances was used. The age gap increases in patients subsequently diagnosed with dementia (mean age: BME patients 78.69 years, s = 6.208, WB patients 83.25 years, s = 6.599; *t*_(187)_ = 4.685, *P*<0.001).

Patients in the BME group scored significantly lower on both the ACE-R and the MMSE:

- ACE-R total possible score 100; mean score: WB patients 66.2, s = 16.652, BME patients 54.5, s = 14.482; *t*_(288)_ = 5.768, *P*<0.001,
- MMSE total possible score 30; mean score: WB patients 23.4, s = 4.917, BME patients 21.7, s = 4.573, *t*_(288)_ = 2.759, *P* = 0.006.

In patients subsequently diagnosed with dementia, there was a significant difference in scores on the ACE-R (WB mean score 57.4, s = 13.464; BME mean score 48.7, s = 11.226; *t*_(187)_ = 4.431, *P*<0.001). BME patients with a new diagnosis of dementia also scored lower on the MMSE, but this difference was not found to be significant (WB mean score 21.0, s = 4.613; BME mean score 20.1, s = 4.166, *t*_(187)_ = 1.294, *P* = 0.197).

Three-quarters of the BME group (75.2%, *n* = 82) were diagnosed with dementia, compared with 65.7% of the WB group (*n* = 157). There was no significant relationship between ethnic group and the likelihood of receiving a diagnosis of dementia (χ^2^_(1, *N* = 348)_ = 2.739, *P* = 0.098). When dementia subtypes were considered, significantly more patients from the BME group were diagnosed with a dementia with a vascular component (either vascular dementia or mixed Alzheimer's disease and vascular dementia) (χ^2^_(1, *N* = 348)_ = 4.531, *P* = 0.033).

## Discussion

Ethnic identity is multifaceted, subjective, can change over time^11^ and may be based on collective identity, common ancestry, heritage, religion, culture, nationality, language and territory.^8^ Individual beliefs may be influenced by culture, and thus culture can shape the meaning which individuals ascribe to dementia.^12^ Beliefs about dementia, such as it being part of normal ageing,^13^ may create barriers to help-seeking and influence when individuals present to memory services.^14^ Current research suggests that BME older people access services at a more severe stage of cognitive impairment than White British people,^15^ which may account for their lower cognitive scores in this study. In BME populations, the decision to seek formal help tends to be precipitated by a health or behavioural crisis^13^ or the emergence of neuropsychiatric symptoms and problems with basic activities of daily living.^4^ It may also be influenced by varying knowledge of Alzheimer's disease and dementia across ethnic groups (although all groups hold significant misperceptions).^5^ All ethnic groups attach stigma to dementia, although the extent and nature of this varies;^5,16^ family concerns about previous negative experiences of psychiatric services may, however, be particularly significant to BME groups.^4^

Cultural bias in cognitive testing may also explain the difference in scores. African–Caribbean patients have consistently been found to score lower than White British patients on the MMSE^17–19^ and the standard cut-off of 24 may have a high rate of false positive screening for dementia in BME groups.^20,21^ Adjusting for factors such as educational attainment did not always explain these differences;^17,22^ scores may also have been affected by unfamiliarity with the language and culture of the test setting, and higher levels of anxiety during testing.^23^ The idea of cultural bias is particularly supported by one study which found that White British participants scored equally well on both the traditional version of the MMSE and a version validated for use with older African–Caribbean people,^24^ while African–Caribbean participants scored significantly better on the culturally modified version.^19^

The ACE-R differs from the MMSE in its greater sensitivity to detect early dementia.^25^ Modifying and validating it for other cultural contexts has required more than straightforward translation, including adaptations to cater for study participants with lower levels of education,^26^ cut-off scores stratified by educational background^27^ and modified cut-offs to account for variations in structure and difficulty between languages.^28^ This demonstrates the difficulty of administering the test to a sample as culturally diverse as that found at the SLMS. Studies validating the ACE-R's diagnostic accuracy fail to mention ethnic diversity within their samples^7,25,29,30^ and tested a clinic-based population with a notably younger mean age than the SLMS sample, therefore their findings may not be transferable. A study carried out in another area of the UK required lower cut-offs to preserve diagnostic accuracy,^31^ although again the ethnic breakdown of participants is unknown.

Most studies carried out in the UK to date have also found a higher prevalence of dementia among African-Caribbean older people,^17,19,32^ although one found differences only between English and non-English-speaking members of BME groups.^33^ The current study found no overall difference in dementia diagnosis rates, but revealed an increased prevalence of dementia with a vascular component in the African–Caribbean group. Although most of the studies cited did not explore dementia subtypes, one linked an increased incidence of conditions such as hypertension and diabetes to a higher prevalence of vascular dementia in their African–Caribbean sample.^17^ Another found an equal prevalence of hypertension in the African–Caribbean and British-born groups, but that awareness of it was reduced among African–Caribbean people, who were significantly more likely to have dementia if hypertension was not correctly treated.^34^ Health education about risk factors for vascular dementia could therefore potentially benefit BME groups, especially given the earlier age of presentation to the SLMS.

Patients access the SLMS through their general practitioner (GP), therefore GPs influence the stage at which patients access specialist help. A suspicion of emerging dementia is often followed by ‘watchful waiting’ in primary care rather than immediate referral to a specialist.^35^ People from BME backgrounds access primary care at a similar rate to White British people,^4,36^ however, there appears to be little research into factors which may influence this wait for specialist referral. Difficulties in cognitive screening in primary care may be further compounded when assessing older BME people, where culture and the language used to describe problems may influence presentation.^22^

### International evidence

This is a UK-based study at a local level, however, its findings are consistent with research carried out abroad. A survey of clinical dementia centres across Europe found varying levels of access by BME patients and that where these patients did access services, diagnostic evaluation was more challenging owing to language barriers and the availability only of cognitive assessment tools validated in Western cultures.^37^ Older Chinese and Vietnamese patients in Australia were believed to present to memory services at a more advanced stage of cognitive impairment^38^ and linguistic and cultural complexities may have contributed to longer waits for diagnosis in primary care.^39^ BME populations were diagnosed with dementia at an earlier age in one Danish study,^40^ and similar barriers to help-seeking for carers of BME patients with dementia were evident in research from both Australia^38,39^ and the USA.^5^ Given the international recognition of the growing need for culturally sensitive memory services (including throughout Europe,^37,40^ the USA^12^ and Australia^38,39^), the findings of this study are likely to be relevant to those working in such settings.

### Limitations

This study is limited in its use of data collected in the course of routine clinical practice. Assessments were carried out by a variety of clinicians and although all were trained in the cognitive tools used, the question of interrater reliability remains. Standardised data on the educational background of participants are also unavailable. In addition, only patients who consented to and completed the assessment and diagnosis process were included in this study. There is no data on the ethnicity of the patients who refused assessment or who were unable to complete the process for other reasons, thus we cannot speculate on whether ethnicity may influence this. Some ethnic groups, such as South Asian people, were not represented in our analysis. The issues influencing presentation to memory services may be similar for these groups; for example, a study on Gujarati populations also found lower MMSE scores and a lower median age in the Gujarati group of a community screening programme.^41^ The small numbers of patients from other BME populations indicates the need for further research into the extent to which they are accessing the SLMS, and limits the ability of this study to draw wider conclusions about the experiences of BME people.

### Clinical implications

Compared with local population figures, the SLMS sample includes a higher proportional representation of African–Caribbean-born people than might be expected. While this finding may seem positive, our results show a more marked degree of cognitive impairment in the BME group, despite the younger age at presentation. This indicates a need to work with local stakeholders to ensure a lower threshold for referring African–Caribbean patients to the service, and to investigate ease of access for patients from other BME groups.

Culturally sensitive assessment is also required. This includes reflection on the way cognitive impairment is assessed in a population with varied cultural and educational backgrounds^42^ and an avoidance of stereotypical beliefs.^43^ Culturally sensitive cognitive tools should be used where they exist, and cognitive testing should not be the only means of determining diagnoses.^29^ The SLMS should continue the approach of considering cognitive test scores in the context of patient and carer accounts, brain neuroimaging, screening for affective disorders and additional neuropsychological testing to ensure accurate diagnosis. GPs also need to be aware of potential differences in age at onset of dementia and vascular risk differentials, to ensure effective cardiovascular preventative measures and arrange appropriate and early onward referral to secondary care.

Further analysis of similar data from a range of local memory services would be useful in detecting whether the trends evident in this study are reflected in the local population and other BME groups. It would be useful to include non-mental health services diagnosing dementia (e.g. geriatricians' clinics and neurology) in this analysis. The analysis into severity of impairment at presentation could be expanded by combining cognitive scores with other rating scales assessing neuropsychiatric symptoms and activities of daily living. Differences in other aspects of service provision and quality of life, such as assessment refusal rates, treatment with cognitive enhancer medications and the role of support networks in encouraging or discouraging help-seeking could also be explored.

Caution must be applied to the interpretation of these results. Although the term BME may imply homogeneity, it encompasses a wide variety of unique individual and collective experiences^9^ and the diversity both within and between ethnic groups must not be overlooked. Ethnicity may play an important role in influencing presentation to memory services, but this is only one part of the picture, and consideration for individual difference must always remain paramount.

## References

[1] Department of Health Equality Impact Assessment: Living Well with Dementia – National Dementia Strategy. Department of Health, 2009 (https://www.gov.uk/government/uploads/system/uploads/attachment_data/file/168222/dh_094054.pdf).

[2] Office for National Statistics Ethnicity and National Identity in England and Wales 2011. ONS, 2012 (http://www.ons.gov.uk/ons/dcp171776_290558.pdf).

[3] Department of Health Living Well with Dementia: A National Dementia Strategy. Department of Health, 2009.

[4] Mukadam N Cooper C Basit B Livingston G Why do ethnic elders present later to UK dementia services? A qualitative study. Int Psychogeriatr 2011; 23: 1070-7. 21349212 10.1017/S1041610211000214

[5] Ayalon L Arean P Knowledge of Alzheimer's disease in four ethnic groups of older adults. Int J Geriatr Psychiatry 2004; 19: 51-7. 14716699 10.1002/gps.1037

[6] Folstein MF Folstein SE McHugh PR Mini-Mental State: A practical method for grading the cognitive state of patients for the clinician. J Psychiatr Res 1975; 12: 189-98. 1202204 10.1016/0022-3956(75)90026-6

[7] Mioshi E Dawson K Mitchell J Arnold R Hodges JR The Addenbrooke's Cognitive Examination Revised (ACE-R): a brief cognitive test battery for dementia screening. Int J Geriatr Psychiatry 2006: 21: 1078-85. 16977673 10.1002/gps.1610

[8] Economic and Social Data Service Ethnicity: Introductory User Guide. ESDS, 2012 (http://www.esds.ac.uk/government/docs/ethnicityintro.pdf).

[9] Manthorpe J Hettiaratchy P Ethnic minority elders in the UK. Int Rev Psychiatry 1993; 5: 171-8.

[10] Office for National Statistics Population Estimates by Ethnic Group: Mid-2009 (experimental). ONS, 2011.

[11] Office for National Statistics Ethnic Group Statistics: A Guide for the Collection and Classification of Ethnicity Data. HMSO, 2003.

[12] Dilworth-Anderson P Gibson B The cultural influence of values, norms, meanings and perceptions in understanding dementia in ethnic minorities. Alzheimer Dis Assoc Disor 2002; 16 (suppl 2s): s56-63. 10.1097/00002093-200200002-00005 12351916

[13] Mukadam N Cooper C Livingston G A systematic review of ethnicity and pathways to care in dementia. Int J Geriatr Psychiatry 2011; 26: 12-20. 21157846 10.1002/gps.2484

[14] La Fontaine J Ahuja J Bradbury NM Phillips S Oyebode JR Understanding dementia amongst people in minority ethnic and cultural groups. J Adv Nurs 2007; 60: 605-14. 18039247 10.1111/j.1365-2648.2007.04444.x

[15] Moriarty J Sharif N Robinson J Research Briefing: Black and Minority Ethnic People with Dementia and Their Access to Support and Services. Social Care Institute for Excellence, 2011.

[16] Lawrence V Samsi K Banerjee S Morgan C Murray J Threat to valued elements of life: the experience of dementia across three ethnic groups. Gerontologist 2011; 51: 39-50. 20724657 10.1093/geront/gnq073

[17] Richards M Brayne C Dening T Abas M Carter J Price M Cognitive function in UK community-dwelling African-Caribbean and White elders: a pilot study. Int J Geriatr Psychiatry 2000; 15: 621-30. 10918343 10.1002/1099-1166(200007)15:7<621::aid-gps164>3.0.co;2-4

[18] Stewart R Johnson J Richards M Brayne C Mann A Medical Research Council Cognitive Functioning and Ageing Study: the distribution of Mini-Mental State Examination scores in an older UK African-Caribbean population compared to MRC CFA study norms. Int J Geriatr Psychiatry 2002; 17: 745-51. 12211125 10.1002/gps.698

[19] Adelman S Blanchard M Rait G Leavey G Livingston G Prevalence of dementia in African-Caribbean compared with UK-born White older people: two-stage cross-sectional study. Br J Psychiatry 2011; 199: 119-25. 21653946 10.1192/bjp.bp.110.086405

[20] Ismail Z Rajji T Shulman K Brief cognitive screening instruments: an update. Int J Geriatr Psychiatry 2010; 25: 111-20. 19582756 10.1002/gps.2306

[21] Fillenbaum G Heyman A Williams K Prosnitz B Burchett B Sensitivity and specificity of standardized screens of cognitive impairment among elderly black and White community residents. J Clin Epidemiol 1990; 43: 651-60. 2370572 10.1016/0895-4356(90)90035-n

[22] Rait G Burns A Screening for depression and cognitive impairment in older people from ethnic minorities. Age Ageing 1998; 27: 271-5.

[23] Parker C Philp I Screening for cognitive impairment among older people in black and minority ethnic groups. Age Ageing 2004; 33: 447-52. 15217776 10.1093/ageing/afh135

[24] Rait G Morley M Burns A Baldwin R Chew-Graham C St-Leger A Screening for cognitive impairment in older African Caribbeans. Psychol Med 2000; 30: 957-63. 11037103 10.1017/s0033291799002305

[25] Mathuranath PS Nestor PJ Berrios GE Rakowicz W Hodges JR A brief cognitive test battery to differentiate Alzheimer's disease and frontotemporal dementia. Neurology 2000; 55: 1613-20. 11113213 10.1212/01.wnl.0000434309.85312.19

[26] Carvalho V Caramelli P Brazilian adaptation of the Addenbrooke's Cognitive Examination-Revised (ACE-R). Dementia Neuropsychologia 2007; 2: 212-6. 10.1590/s1980-57642008dn10200015 PMC5619571 29213390

[27] Mathuranath PS Cherian JP Mathew R George A Alexander A Sarma SP Mini Mental State Examination and the Addenbrooke's Cognitive Examination: effect of education and norms for a multicultural population. Neurol India 2007; 55: 106-10. 17558112 10.4103/0028-3886.32779

[28] Yoshida H Terada S Honda H Ata A Takeda N Kishimoto Y Validation of Addenbrooke's cognitive examination for detecting early dementia in a Japanese population. Psychiatry Res 2011; 185: 211-4. 20537725 10.1016/j.psychres.2009.06.012

[29] Crawford S Whitnall L Robertson J Evans J A systematic review of the accuracy and clinical utility of the Addenbrooke's Cognitive Examination and the Addenbrooke's Cognitive Exmination-Revised in the diagnosis of dementia. Int J Geriatr Psychiatry 2012; 27: 659-69. 22068971 10.1002/gps.2771

[30] Dudas R Berrios G Hodges J The Addenbrooke's Cognitive Examination (ACE) in the differential diagnosis of early dementia versus affective disorder. Am J Geriatr Psychiatry 2005; 13: 218-26. 15728753 10.1176/appi.ajgp.13.3.218

[31] Larner AJ Addenbrooke's Cognitive Examination (ACE) for the diagnosis and differential diagnosis of dementia. Clin Neurol Neurosurg 2007; 109: 491-4. 17509752 10.1016/j.clineuro.2007.04.004

[32] Livingston G Leavey G Kitchen G Manela M Sembhi S Katona C Mental health of migrant elders – the Islington study. Br J Psychiatry 2001; 179: 361-6. 11581119 10.1192/bjp.179.4.361

[33] McCracken CF Boneham MA Copeland JR Williams KE Wilson K Scott A Prevalence of dementia and depression among elderly people in black and ethnic minorities. Br J Psychiatry 1997; 171: 269-73. 9337983 10.1192/bjp.171.3.269

[34] Stevens T Leavey G Livingston G Dementia and hypertension in African/Caribbean elders. Age Ageing 2004; 33: 193-5. 14960438 10.1093/ageing/afh042

[35] Iliffe S Robinson L Brayne C Goodman C Rait G Manthorpe J Primary care and dementia: 1. Diagnosis, screening and disclosure. Int J Geriatr Psychiatry 2009; 24: 895-901. 19226529 10.1002/gps.2204

[36] Richards M Brayne C Forde C Abas M Levy R Surveying African Caribbean elders in the community: implications for research on health and health service use. Int J Geriatr Psychiatry 1996; 11: 41-5.

[37] Nielsen T Vogel A Riepe M de Mendonca A Rodriguez G Nobili F Assessment of dementia in ethnic minority patients in Europe: a European Alzheimer's disease Consortium survey. Int Psychogeriatr 2011; 23: 86-95. 20602861 10.1017/S1041610210000955

[38] Haralambous B Dow B Tinney J Lin X Blackberry I Rayner V Help seeking in older Asian people with dementia in Melbourne: using the Cultural Exchange Model to explore barriers and enablers. J Cross Cult Gerontol 2014; 29: 69-86. 24443007 10.1007/s10823-014-9222-0

[39] Lee S Lin X Haralambous B Vrantsidis F Tinney J Blackberry I Factors impacting on early detection of dementia in older people of Asian background in primary healthcare. Asia-Pacific Psychiatry 2011; 3: 120-7.

[40] Nielsen T Vogel A Phung T Gade A Waldemar G Over- and under-diagnosis of dementia in ethnic minorities: a nationwide register-based study. Int J Geriatr Psychiatry 2011; 3: 1128-35. 21194100 10.1002/gps.2650

[41] Lindesay J Jagger C Mlynij-Szmid A Sinorwala A Peet S Moledina F The Mini-Mental State Examination (MMSE) in an elderly immigrant Gujarati population in the United Kingdom. Int J Geriatr Psychiatry 1997; 12: 1155-67. 9444539

[42] Daker-White G Beattie AM Gilliard J Means R Minority ethnic groups in dementia care: a review of service needs, service provision and models of good practice. Aging Ment Health 2002; 6: 101-8. 12028878 10.1080/13607860220126835

[43] Moriary J Better Health Briefing 9: The Health and Social Care Experiences of Black and Ethnic Minority Older People. Race Equality Foundation, 2008.

